# Supplementary material for: Nuquantus: Machine learning software for the characterization and quantification of cell nuclei in complex immunofluorescent tissue images
Source: Sci Rep. 2016 Mar 23;6:23431. doi: 10.1038/srep23431 (PMC4804284; doi:10.1038/srep23431)
Supplement: Supplementary Information [file srep23431-s1.pdf]

Nuquantus: Machine learning software for the characterization and quantification of cell nuclei in complex immunofluorescent tissue images

Polina Gross[1,+], Nicolas Honnorat[2,+], Erdem Varol[2,+], Markus Wallner[1], Danielle M. Trappanese[1], Thomas E. Sharp[1], Timothy Starosta[1], Jason M. Duran[1], Sarah Koller[1], Christos Davatzikos[2*] and Steven R. Houser[1*]

[1] Temple University School of Medicine, Cardiovascular Research Center, Department of Physiology, Philadelphia, PA 19140, USA.

[2] University of Pennsylvania, Center for Biomedical Image Computing and Analytics, Department of Radiology, Philadelphia, PA, 19104, USA.

[*] Corresponding authors (Christos.Davatzikos@uphs.upenn.edu and srhouser@temple.edu)
[+] These authors contributed equally to this work

SUPPLEMENTARY MATERIAL

METHODS

The following parameters were used for feature extraction:

1. Fourier HOG at bin size 6 and scale 8.
2. Haralick features were computed within a patch of 15 pixels radius in the red channel (α-sarcomeric actin/tropomyosin staining).
3. Entropy was computed within a square 9X9 patch of the composite red and blue channel image (α-sarcomeric actin/tropomyosin and DAPI staining).
4. The cell membranes were extracted from the red channel by applying the steerable filters[1] at scales [1.0,1.25,1.5,1.75,2.0,5.0,7.5,10.0,15.0]. The output of these filters were combined into a unique membrane map following[2]. Tensor Voting method[3] for 10, 8, and 6 scales was finally used for improving the output of the detector.
5. Extracellular pixels were defined as those with intensity less than 40. The binary map of extracellular pixels was dilated by 1, 2 and 3 pixels. Connected components were extracted and their size was reported into each pixel part of the component. Measuring these sizes allows discriminating the interstitial tissue from the large extracellular compartments, which greatly helps discarding extra tissue nuclei. Example: Extracellular component size (ECS) is equal to 0 when the pixel is in cardiac tissue. Otherwise, ECS is greater than 0.

Other state-of-the art features were tested, such as Gabor filters, the size of the nuclei, different color representations and different image scales. They appeared to be redundant with our current set of features and were removed for the sake of simplicity.

FIGURE LEGENDS

Supplemental Figure S1. Fluorescence Image preprocessing for intensity correction. a. Original confocal microscopy image of cardiac tissue that was fluorescently stained for α-sarcomeric actin and DAPI. **b.** The original image is processed using Gaussian blur filter (scale of 10 pixels to the red channel representing α-actin/tropomyosin staining) to compute the average brightness around each pixel. This computation illustrates heterogeneous color and illumination artifacts that may occur due to variation in staining and confocal laser scanning. **c.** Illumination artifacts are corrected by normalizing the average brightness such that the maximum across the image is 1. Each pixel intensity value was divided by the normalized average intensity of its neighborhood. **d.** The corrected image is visualized via Gaussian blur filter to visualize the pixels that were bright enough before the correction remain unchanged, while the dim pixels are enhanced. Scale 20 µm.

Supplemental Figure S2. Histogram of probability scores given to nuclei. Distribution of CM and non-CM nuclei based on probabilities scores for **a.** swine prediction model and. **b.** mouse predication model. Each nucleus was screened by the Nuquantus prediction model and assign with a probability score. Higher probability score (>0.2) provides prediction of prospective non-CM nucleus. Lower probability score (<0.2) classifies the nucleus as a CM nucleus.

Supplemental Figure S3. Nuquantus validation for EdU and DAPI positive nuclei count.

The nuclei in 48 images obtained from post MI mice (N=4) and control shams (N=4) were counted twice: manually and then with Nuquantus software. The MI cardiac sections were analyzed for IA, BZ and VZ (3 images per sub-region per animal). Shams cardiac sections were analyzed using 3 images per animal similarly to VZ. The total nuclei count and CM nuclei count undergoing DNA synthesis (EdU + DAPI) that was measured by Nuquantus was compared to the manual counts. Trends showed matching counts without any statistical significant difference. (Non-parametric paired Wilcoxon test).

Supplemental Figure S4. Nuquantus classifies nuclei of different cell subtypes undergoing DNA synthesis. a. Example of IA image that was analyzed by Nuquantus. **a1.** Original image. **a2-a7.** Nuquantus output of analyzed IA image after image preprocessing and user correction step. **a2**. Total of 393 nuclei (CM + non-CM nuclei) labeled with DAPI. **a3.** Total of 279 cellular components (CM + non-CM nuclei) labeled with EdU. **a4.** 385 non CM nuclei labeled with DAPI. **a5.** Total of 218 nuclei (CM + non-CM nuclei) co-labeled with DAPI and EdU. **a6.** Eight CM nuclei labeled with DAPI. **a7.** Two CM nuclei co-labeled with DAPI and EdU. **b.** Example of analyzed BZ image. **b1.** Original image. **b2-b7.** Nuquantus output. **b2**. Total of 304 nuclei (CM + non-CM nuclei) labeled with DAPI. **b3.** Total of 297 cellular components (CM + non-CM nuclei) labeled with EdU. **b4.** 290 non CM nuclei labeled with DAPI. **b5.** Total of 196 nuclei (CM + non-CM nuclei) co-labeled with DAPI and EdU. **b6.** 14 CM nuclei labeled with DAPI. **b7.** Three CM nuclei co-labeled with DAPI and EdU. **c.** Example of analyzed VZ image. **c1.** Original image. **c2-c7.** Nuquantus output. **c2**. Total of 327 nuclei (CM + non-CM nuclei) labeled with DAPI. **c3.** Total of 193 cellular components (CM + non-CM nuclei) labeled with EdU. **c4.** 287 non CM nuclei labeled with DAPI. **c5.** Total of 167 nuclei (CM + non-CM nuclei) co-labeled with DAPI and EdU. **c6.** 40 CM nuclei labeled with DAPI. **c7.** Four CM nuclei co-labeled with DAPI and EdU.
Note that cases in which few segmented EdU labels co-localized with one nucleus identified by DAPI, were considered as a single EdU + DAPI nucleus. Scale 20µm.

Supplemental Figure S5. Nuquantus validation for TUNEL and DAPI positive nuclei count. 30 images obtained from N=4 healthy mice were analyzed for nuclei total count, total TUNEL positive nuclei count, CM nuclei count and TUNEL positive CM nuclei count. The counts were compared between negative and positive TUNEL controls using manual approach and Nuquantus software with applied user correction step. No statistical significant difference was detected by a non-parametric paired Wilcoxon test.

SUPPLEMENTARY REFERENCES

1. Jacob, M. & Unser, M. Design of steerable filters for feature detection using canny-like criteria. *Pattern Analysis and Machine Intelligence, IEEE Transactions on* **26**, 1007-1019 (2004).
2. Lindeberg, T. Edge detection and ridge detection with automatic scale selection. *International Journal of Computer Vision* **30**, 117-156 (1998).
3. Franken, E., van Almsick, M., Rongen, P., Florack, L. & ter Haar Romeny, B. An efficient method for tensor voting using steerable filters, in *Computer Vision–ECCV 2006* 228-240 (Springer, 2006).

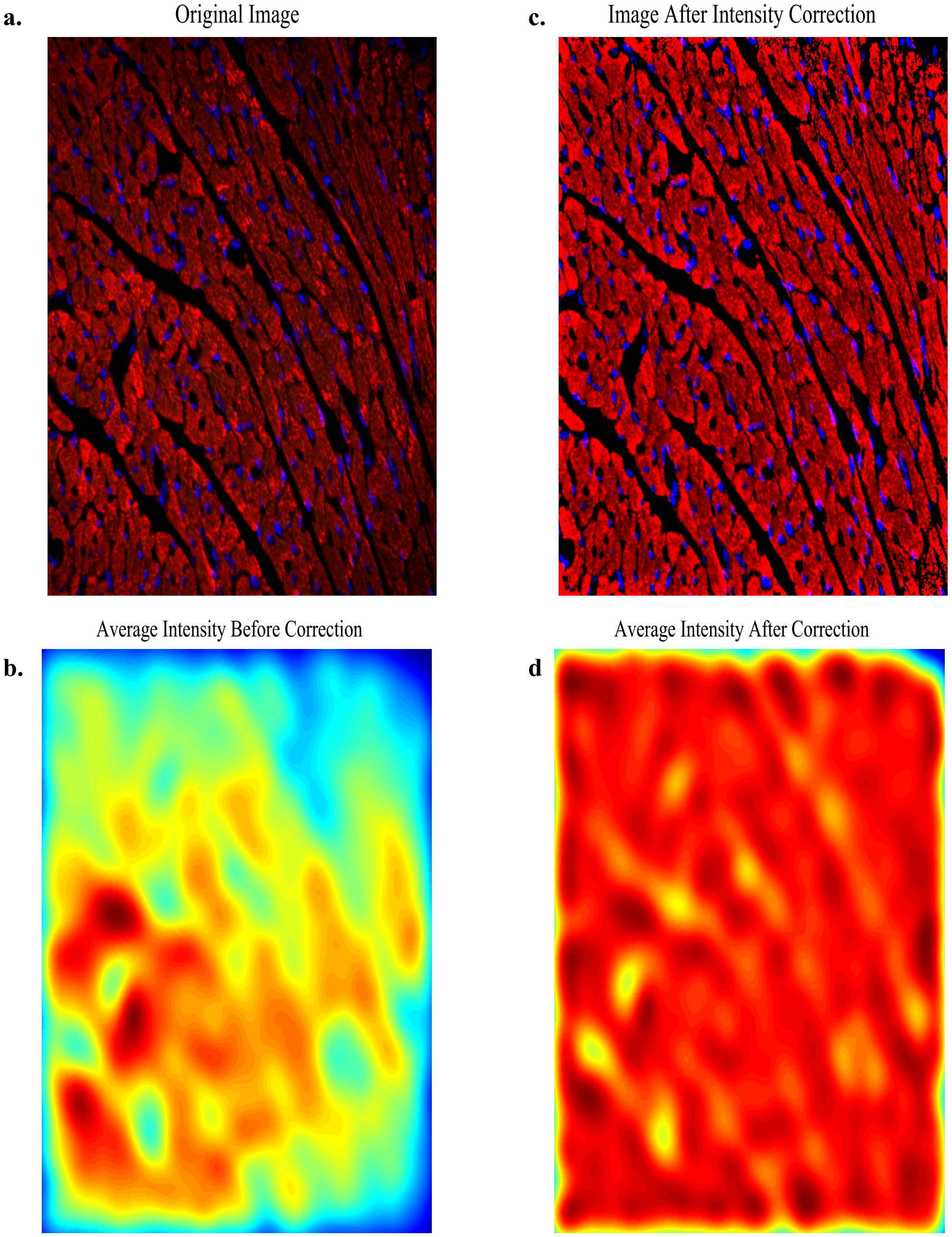

Supplementary Figure 1

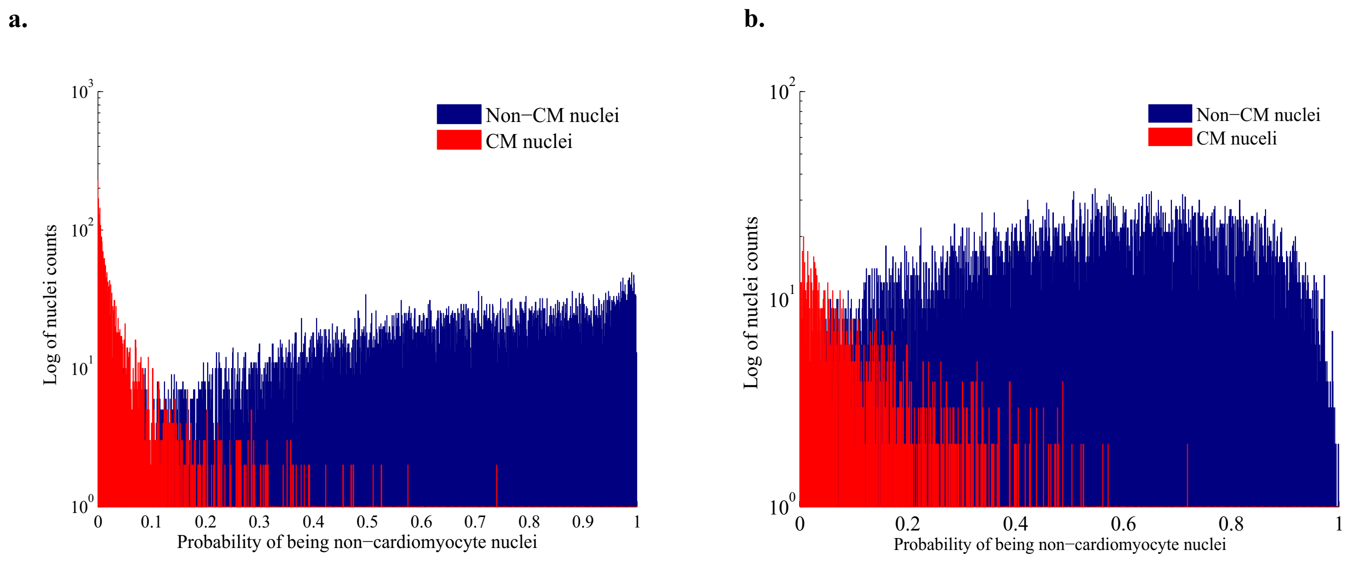

Supplementary Figure 2

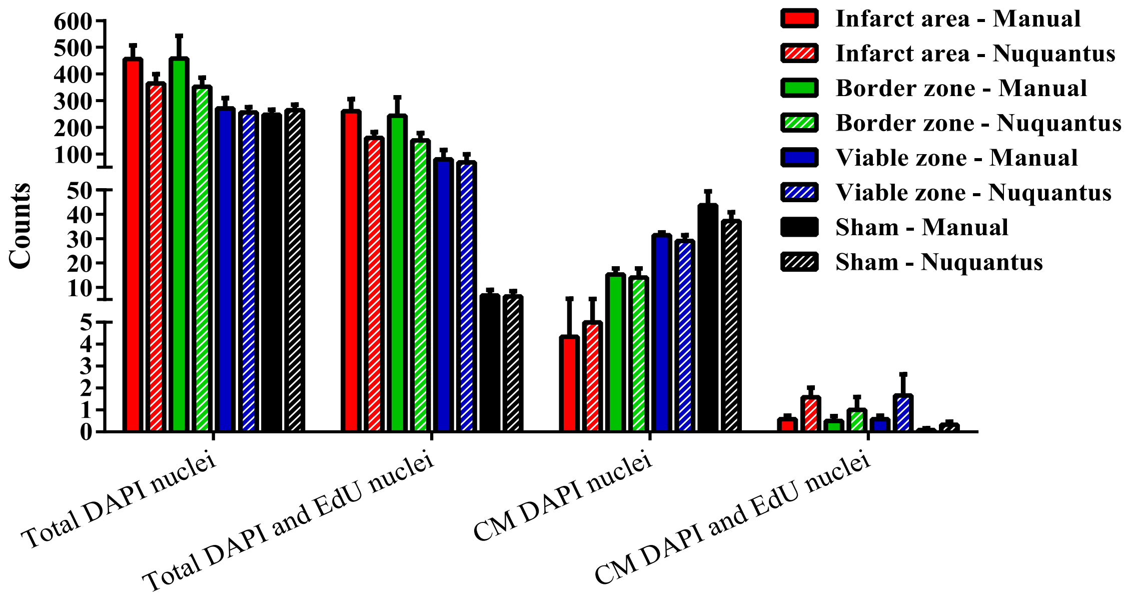

Supplementary Figure 3

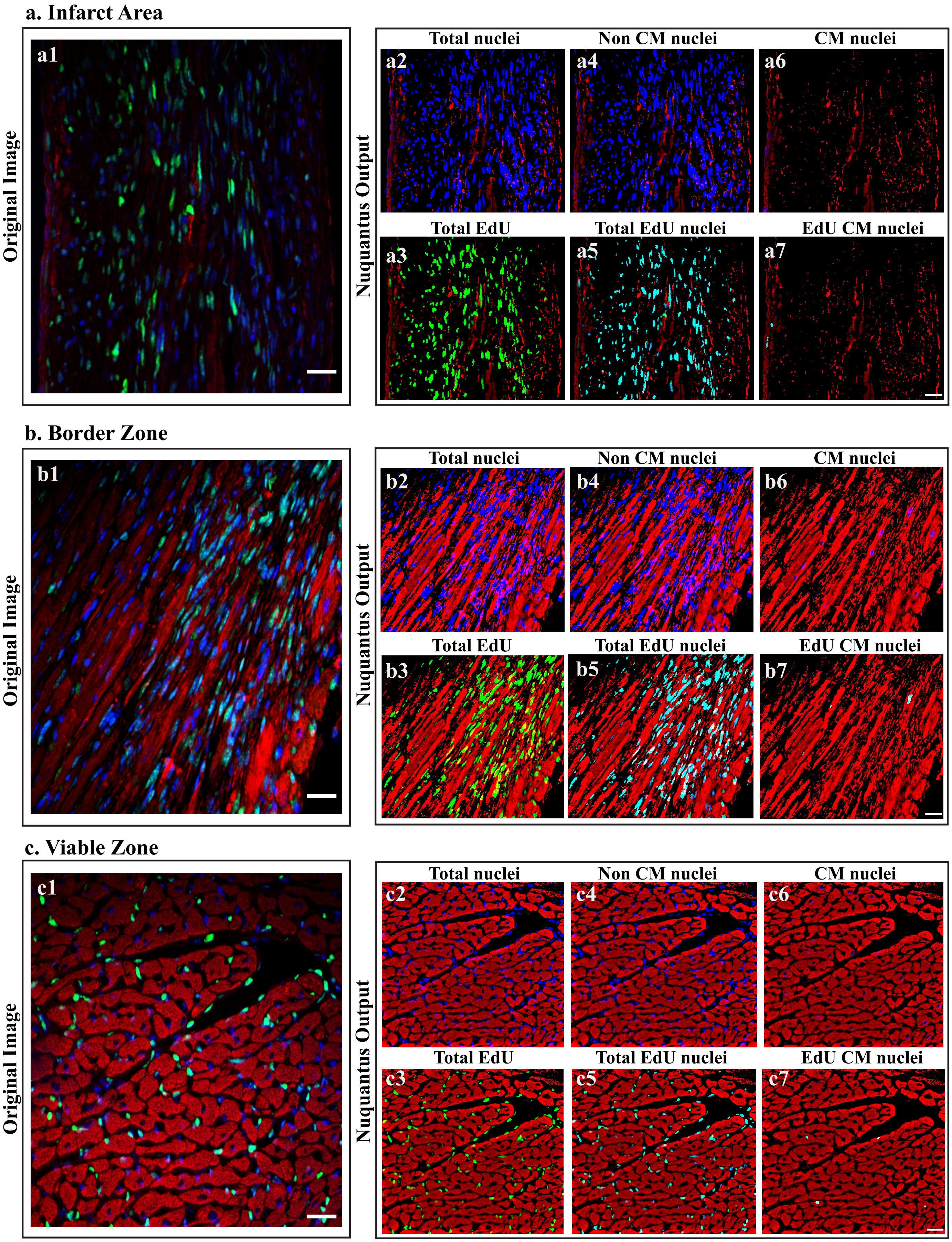

Supplementary Figure 4

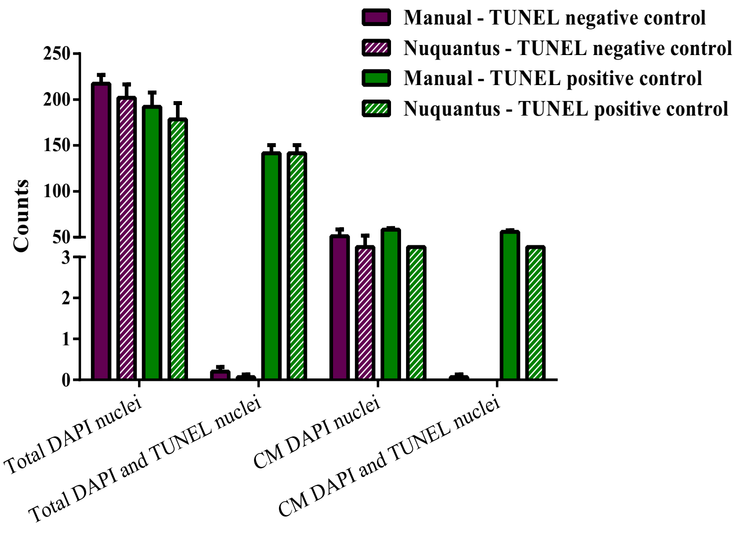

Supplementary Figure 5
